# Differential Dynamics of Regulatory T-Cell and Th17 Cell Balance in Mesenteric Lymph Nodes and Blood following Early Antiretroviral Initiation during Acute Simian Immunodeficiency Virus Infection

**DOI:** 10.1128/JVI.00371-19

**Published:** 2019-09-12

**Authors:** Alexis Yero, Omar Farnos, Henintsoa Rabezanahary, Gina Racine, Jérôme Estaquier, Mohammad-Ali Jenabian

**Affiliations:** a Department of Biological Sciences, Université du Québec à Montréal (UQAM), Montreal, Quebec, Canada; b Centre Hospitalier Universitaire (CHU) de Québec Research Center, Faculty of Medicine, Université Laval, Quebec City, Quebec, Canada; Emory University

**Keywords:** antiretroviral therapy, early antiretroviral therapy, gut mucosal immunity, human immunodeficiency virus, immune dysfunction, mucosal immunity, regulatory T cells, Tregs, simian immunodeficiency virus, Th17 cells

## Abstract

Increased frequencies of immunosuppressive regulatory T cells (Tregs) are associated with gut lymphoid tissue fibrosis and dysfunction which, in turn, contribute to disease progression in chronic simian immunodeficiency virus/human immunodeficiency virus (SIV/HIV) infection. Mesenteric lymph nodes (MLNs), which drain the large and small intestine, are critical sites for the induction and maintenance of gut mucosal immunity. However, the dynamics of Tregs in MLNs are not well understood due to the lack of accessibility to these tissues in HIV-infected individuals. Here, the dynamics of Tregs in blood and MLNs were assessed in SIV-infected rhesus macaques (RMs) following early antiretroviral drug (ARV) initiation. Early ARV initiation reduced T-cell immune activation, as assessed by HLA-DR/CD39 expression, and prevented the depletion of memory CCR6^+^ Th17 cells in both blood and MLNs. Untreated animals showed higher frequencies of Tregs, CD39^+^ Tregs, thymic Tregs, and new memory CD4 populations sharing similarity with Tregs as CTLA4^+^ PD1^–^ and CTLA4^+^ PD1^–^ FoxP3^+^ T cells. Despite early ARV treatment, the frequencies of these Treg subsets remained unchanged within the MLNs and, in contrast to blood normalization, the Th17/Treg ratio remained distorted in MLNs. Furthermore, our results highlighted that the expressions of IDO-1, TGFβ1 and collagen-1 mRNA remained unchanged in MLN of ARV-treated RMs. ARV interruption did not affect T-cell immune activation and Th17/Treg ratios in MLN. Altogether, our data demonstrated that early ARV initiation within the first few days of SIV infection is unable to reduce the frequencies and homing of various subsets of Tregs within the MLNs which, in turn, may result in tissue fibrosis, impairment in MLN function, and HIV persistence.

**IMPORTANCE** Tregs contribute to SIV/HIV disease progression by inhibition of antiviral specific responses and effector T-cell proliferation. Tregs also cause tissue fibrosis via transforming growth factor β1 production and collagen deposition, which are associated with microbial translocation and generalized immune activation. Early ARV initiation upon viral exposure is recommended globally and results in improved immune function recovery and reduced viral persistence. Here, using an acute SIV infection model of rhesus macaques, we demonstrated for the first time that despite clear improvements in mucosal CD4 T cells, in contrast to blood, Treg frequencies in MLNs remained elevated following early ARV initiation. The particular Th17/Treg balance observed in MLNs can contribute, in part, to the maintenance of mucosal fibrosis during suppressive ARV treatment. Our results provide a better understanding of gut mucosal immune dynamics following early ARV initiation. These findings suggest that Treg-based treatments could serve as a novel immunotherapeutic approach to decrease gut mucosal damage during SIV/HIV infections.

## Abstract (precis)

Tregs contribute to SIV/HIV disease progression by inhibition of antiviral specific responses and effector T-cell proliferation. Tregs also cause tissue fibrosis via transforming growth factor β1 production and collagen deposition, which are associated with microbial translocation and generalized immune activation. Early ARV initiation upon viral exposure is recommended globally and results in improved immune function recovery and reduced viral persistence. Here, using an acute SIV infection model of rhesus macaques, we demonstrated for the first time that despite clear improvements in mucosal CD4 T cells, in contrast to blood, Treg frequencies in MLNs remained elevated following early ARV initiation. The particular Th17/Treg balance observed in MLNs can contribute, in part, to the maintenance of mucosal fibrosis during suppressive ARV treatment. Our results provide a better understanding of gut mucosal immune dynamics following early ARV initiation. These findings suggest that Treg-based treatments could serve as a novel immunotherapeutic approach to decrease gut mucosal damage during SIV/HIV infections.

## INTRODUCTION

Simian immunodeficiency virus/human immunodeficiency virus (SIV/HIV) infections are characterized by rapid CD4 T-cell depletion and death by apoptosis, increased immune activation, and dysfunction of antigen-specific T cells starting during the acute infection (1–3). One of the main sites of SIV/HIV replication and CD4 T-cell depletion is the gut-associated lymphoid tissues (GALT) (2, 4–6). Within mucosal CD4 T-cell subsets, memory CCR6^+^ Th17 cells become depleted very early after acute SIV/HIV infection which results in GALT dysfunction (7–10) since these cells play a crucial role in maintaining of gut mucosal immunity due to the secretion of interleukin-17 (IL-17) (5, 11, 12).

Regulatory T cells (Tregs) constitute a subset of CD4^+^ T cells, commonly characterized by the expression of transcription factor FoxP3, which are immunosuppressive cells playing a pivotal role in the control of immune tolerance and autoimmune diseases. Tregs also contribute to the limitation of immune activation and T-cell function in cancer or chronic viral infections (13, 14). Furthermore, Tregs can be classified into natural or thymic Tregs and induced or adaptive Tregs based on their differentiation within the thymus and inflammatory tissues, respectively. Indeed, thymic Tregs are generated in the thymus as a distinct T-cell lineage, whereas induced Tregs (iTregs) are differentiated from FoxP3^–^ conventional CD4^+^ T cells, which become FoxP3^+^ in the periphery during inflammation (14). It has been shown by us and other groups that SIV/HIV infections result in an increase in Treg activation and frequencies in both acute and chronic infections (13–16), and these cells are involved in disease progression as Tregs inhibit HIV-specific responses, T-cell proliferation, and cytokine production, thus contributing to viral persistence (13–15, 17). Importantly, GALT dysfunction due to the imbalance between Tregs and mucosal CCR6^+^ Th17 cells in favor of Tregs may contribute in the breakdown of mucosal immunity, leading to microbial translocation and systemic immune activation during SIV/HIV infections (2, 18–20). Interestingly, a new subset of memory CD4 T cells, described as CTLA4^+^ PD1^–^, which shares partially Treg phenotypic features contributes to SIV persistence (21), whereas their dynamic overtime has not yet been fully documented.

It is well documented that Tregs promote collagen deposition and subsequent mucosal fibrosis via the secretion of transforming growth factor β1 (TGF-β1) starting from the acute phases of SIV/HIV infections particularly in mesenteric lymph nodes (MLNs), which drain the small and large intestines (4, 22), and in lower levels in nonpathogenic lentiviral infections such as in African green monkeys (22). Tissue fibrosis impedes cell-cell interactions, limits normal traffic and homing of CD4 T cells, and decreases the survival of resident cells by decreasing their access to the cytokines such as IL-2 and IL-7 (23). Interestingly, a low frequency of peripheral Tregs in the blood of HIV elite controllers who spontaneously control viral replication may contribute to effective adaptive immune responses (24), and experimental Treg depletion in SIV-infected controller macaques resulted in higher SIV-specific CD8^+^ T-cell frequencies (25). However, GALT fibrosis occurs even in HIV long-term nonprogressors and elite controllers, suggesting that the dynamics of Tregs could be distinct in the GALT compared to the blood (26).

The initiation of antiretroviral drugs (ARV) upon HIV exposure is now recommended globally in clinical practice since this results in a better CD4 T-cell recovery and disease outcomes and, importantly, smaller viral reservoir sizes (2, 27). However, ARV failed to completely reverse HIV-induced damaged in GALT and to eradicate the infection, even after very early ARV initiation (2, 26, 28). We previously showed that long-term ARV may normalize Treg frequencies in the blood at a similar level to that observed for uninfected individuals (19). However, to date, Treg dynamics during acute infection and the impact of early ARV initiation on Tregs, especially in the MLNs, which are crucial for maintaining gut mucosal immune responses, remains understudied. We therefore assessed here the dynamic of Tregs in these tissues of SIV-infected rhesus macaques (RMs) after early ARV initiation.

## RESULTS

### Early ARV initiation results in total CD4 T-cell recovery and decreased levels of T-cell immune activation in both blood and MLNs.

**FIG 1:**
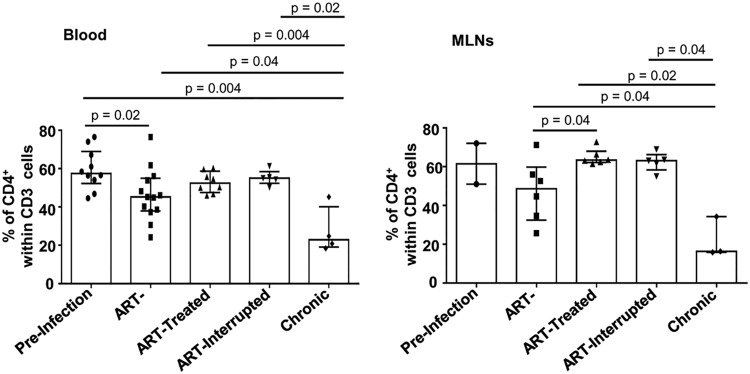
Effect of early ARV initiation on CD4 T-cell recovery. The frequencies of CD4 T cells within CD3 cells in blood (A) and MLNs (B) were determined.

**FIG 2:**
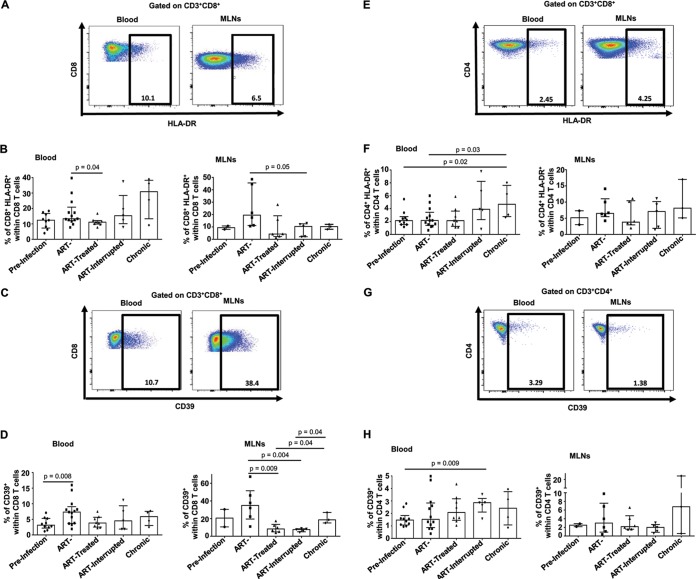
Effect of early ARV initiation on immune activation. (A and E) Gating strategy used in flow cytometry to define HLA-DR^+^ CD8 and CD4 T cells in both whole blood and MLNs. (B and F) Percentages of activated HLA-DR^+^ CD8 and CD4 T cells among total CD8 and CD4 T cells in whole blood and MLNs. (C and G) Gating strategy used in flow cytometry to define CD39^+^ CD8 and CD4 T cells in both whole blood and MLNs. (D and H) Percentages of activated CD39^+^ CD8 and CD4 T cells among total CD8 and CD4 T cells in whole blood and MLNs.

We recently demonstrated an early depletion of memory CD4 T cells in MLNs (29) and, as expected, our results showed that early ARV treatment preserves CD4 T cells in both blood and MLNs (Fig. 1). Since SIV/HIV infections promote T-cell immune activation (8, 30), we then evaluated the effect of early ARV initiation on T-cell immune activation by assessing the expression of HLA-DR and CD39, an ectonucleotidase that converts ATP into immunosuppressive adenosine in concert with CD73, both known as markers of T-cell activation (13, 31–33). Consistent with the drastic reduction of plasma viral load in ARV-treated RMs (Table 1), we observed a significant decrease in CD8 T cells expressing HLA-DR (Fig. 2A and B) and CD39 (Fig. 2C and D), both in peripheral blood and MLNs, compared to untreated animals in acute and chronic phases. Interestingly, despite a rebound in viremia after ARV interruption, the level of CD8 T-cell immune activation remains low both in the blood and MLNs. Similarly, HLA-DR expression by CD4 T cells in MLNs followed the same tendency as CD8 T cells described above, whereas no change was observed in frequencies of CD4^+^ CD39^+^ in the MLNs of different study groups (Fig. 2E to H). Altogether, these results demonstrate that early ARV initiation decreases T-cell immune activation in both blood and MLNs.

**TABLE 1:** Description of animals in each study group^*a*^

SIV status	Group	Monkey ID	Plasma viral load (copies/ml)	Days of infection	Blood	MLNs
SIV^–^	Uninfected	PB030	NA	NA	+	–
		PB033	NA	NA	+	–
		PB036	NA	NA	+	–
		PB046	NA	NA	+	–
		PB049	NA	NA	+	–
		PB051	NA	NA	+	–
		PB061	NA	NA	+	–
		PB057	NA	NA	+	–
		9052732	NA	NA	+	+
		9071222	NA	NA	+	+
						
SIV^+^	Nontreated (acute phase)	PB006	2.8 × 10^7^	11	+	–
		PB041	1.9 × 10^6^	11	+	–
		PB005	5.8 × 10^6^	14	+	–
		PB051	4.7 × 10^7^	14	+	–
		PB015	1.1 × 10^6^	29	+	+
		PB033	6.5 × 10^6^	29	+	+
		9051222	1.6 × 10^6^	33	+	+
		PB044	7.1 × 10^5^	33	+	–
		PB023	5.7 × 10^6^	36	+	–
		PB028	2.3 × 10^6^	36	+	–
		PB055	3.2 × 10^7^	36	+	+
		PB030	2.1 × 10^7^	46	+	+
		9082012	1.7 × 10^4^	60	+	+
	ART-treated (acute phase)	R110806	5.8 × 10^2^	11	+	+
		11-1466R	ND	14	+	+
		R110804	ND	14	+	–
		R110562	ND	27	+	+
		11-1430R	ND	28	+	–
		R110360	ND	35	+	+
		R110482	ND	35	+	–
		13-1660R	ND	36	–	+
		12-1836R	ND	55	+	+
	ART-interrupted (acute phase)	R110482	1.1 × 10^5^	72	+	+
		12-1888R	3.7 × 10^3^	72	+	+
		R110804	3.1 × 10^5^	75	+	+
		12-1134R	7.6 × 10^5^	75	+	+
		11-1430R	3.5 × 10^7^	78	+	+
	Nontreated (chronic phase)	PB023	1.4 × 10^8^	167	+	+
		PB013	3.6 × 10^6^	188	+	+
		PB028	1.6 × 10^8^	194	+	–
		PB044	5.7 × 10^7^	223	+	+

a NA, not applicable; ND, not detectable. Blood specimens from three ART-treated animals were assessed longitudinally in preinfection and acute phase (monkey IDs PB030, PB033, and PB051), and three other ART-treated animals were assessed longitudinally in both acute and chronic phases (monkey IDs PB044, PB028, and PB023).

### Early ARV initiation restores levels of memory CCR6^+^ Th17 cells in both blood and MLNs.

**FIG 3:**
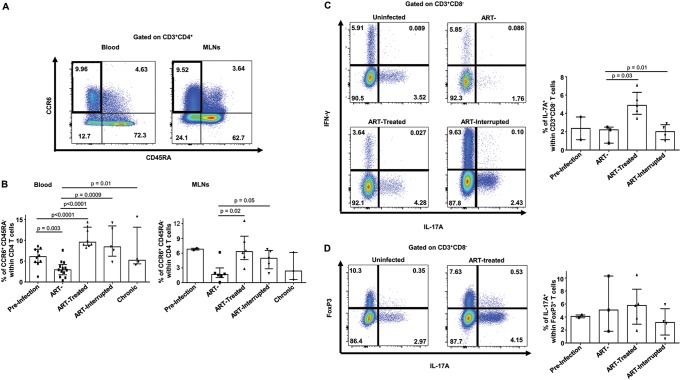
Effect of early ARV initiation on memory CCR6^+^ Th17 CD4 T cells. (A) Gating strategy used in flow cytometry to identify CCR6^+^ memory CD4 T cells in both whole blood and MLNs. (B) Percentages of CCR6^+^ memory CD4 T cells among total CD4^+^ T cells in whole blood and MLNs. (C) IL-17a and IFN-γ expression in T cells of MLNs cells from early ARV-treated animals following PMA/ionomycin stimulation. (D) IL-17a expression by CD4^+^ FoxP3^+^ cells in MLNs from early ARV-treated animals following PMA/ionomycin stimulation.

It is well documented that a subset of Th17 cells, known as memory CCR6^+^ T cells, are depleted rapidly following SIV/HIV infections (5, 9, 11). We therefore assessed the effect of early ARV treatment on the dynamics of memory CCR6^+^ Th17 cells. As expected, a rapid decline has been observed in peripheral memory CCR6^+^ Th17 cells in untreated RMs starting from the acute phase of SIV infection (Fig. 3A and B). Accordingly, these cells are also low in MLNs of untreated RMs in both acute and chronic phases. Importantly, early ARV treatment resulted in significant memory CCR6^+^ Th17 cell recovery compared to untreated SIV-infected RMs in acute and chronic phases in both compartments. Interestingly, the levels of this population in the blood were even higher in ARV-treated RMs compared to healthy RMs and remain elevated in RMs in which ARV has been interrupted (Fig. 3B). To functionally confirm these data, we measured the *in vitro* production of IL-17a in cells isolated from the MLNs. We observed, here again, a decline in IL-17a-expressing CD4 T cells following acute SIV infection which was recovered by early ARV (Fig. 3C). We observed higher levels of IL-17a-expressing cells in ARV-treated RMs compared to untreated SIV-infected RMs and, of importance, our results demonstrated a drastic decrease in IL-17a-expressing CD4 T cells after ARV interruption (Fig. 3C). Since the contribution of IL-17a-expressing CD4^+^ FoxP3^+^ in inflammatory intestinal diseases has been previously reported (34), we also assessed the expression of IL-17 in these cells, as shown in Fig. 3D. A very small proportion of CD4^+^ FoxP3^+^ cells produced IL-17a, a level which remains similar in the different study groups. Altogether, these results demonstrate that early ARV initiation preserves memory CCR6^+^ Th17 cells.

### Treg frequencies in MLNs remain unchanged despite early ARV initiation.

**FIG 4:**
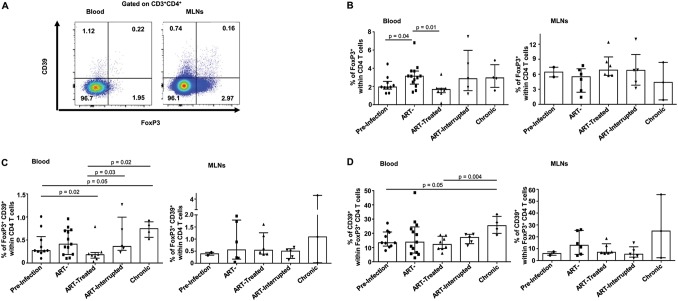
Effect of early ARV initiation on Treg dynamics. (A) Gating strategy used in flow cytometry to define total Tregs and Tregs expressing CD39 in both whole blood and MLNs. (B) Percentages of FoxP3^+^ CD4 T cells among total CD4 T cells in whole blood and MLNs. (C) Percentages of CD39^+^ FoxP3^+^ CD4 T cells among total CD4 T cells in whole blood and MLNs. (D) Percentages of CD39^+^ cells among FoxP3^+^ CD4 T cells in whole blood and MLNs.

Both acute and chronic SIV/HIV infections are associated with increases in total Treg (CD4^+^ Foxp3^+^) and CD39^+^ Treg (CD4^+^ Foxp3^+^ CD39^+^) frequencies in peripheral blood and GALT of untreated individuals which contribute to a poor effector T-cell-specific immune response, GALT fibrosis, and dysfunction and disease progression (4, 13, 14, 23, 33, 35, 36). Here, we observed that the frequencies of Tregs and of CD39^+^ Tregs in the blood of SIV-infected RMs increased during both acute and chronic phases of infection (Fig. 4). Early ARV treatment decreased the frequencies of blood Tregs and CD39^+^ Tregs compared to acutely SIV-infected RMs. Paradoxically, compared to blood, our results indicated that early ARV treatment did not decrease the frequencies of Tregs and CD39^+^ Tregs within MLNs (Fig. 4B to D).

**FIG 5:**
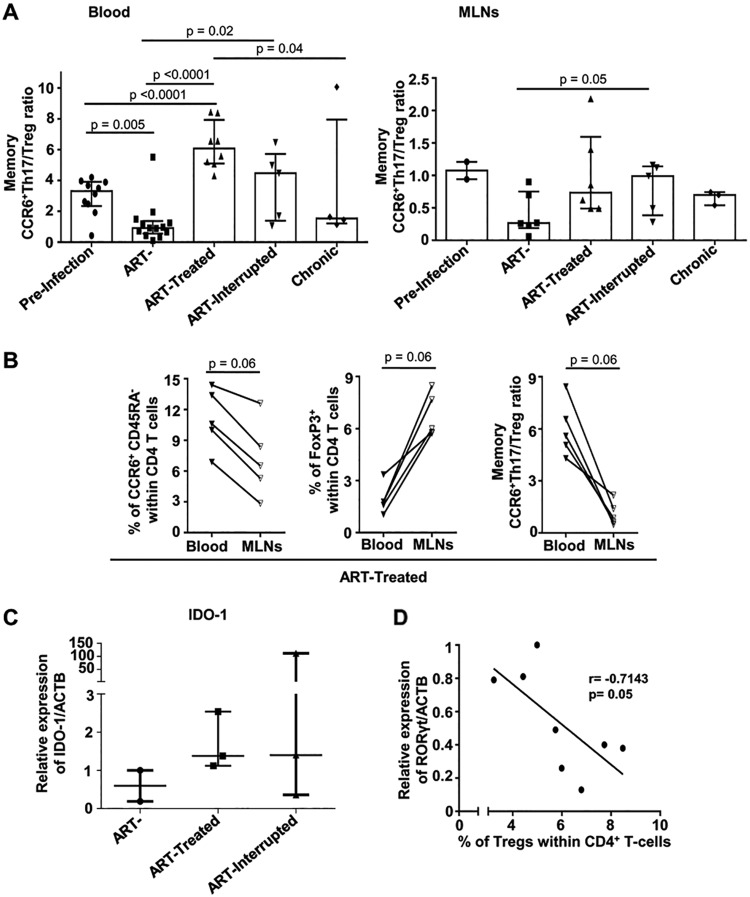
Effect of early ARV initiation on CCR6^+^ Th17/Treg ratio. (A) Memory CCR6^+^ Th17/Treg ratio in both whole blood and MLNs. (B) Differential dynamics of Tregs and Th17 cells in matched blood versus MLNs of early ARV-treated animals. (C) Relative expression of IDO-1 mRNA relative to the ACTB housekeeping gene in a subset of animals for which the snap-frozen MLN tissues was available (*n* = 8). (D) Correlation between RORγt mRNA expression and frequencies of total FoxP3^+^ with the MLNs.

The Th17/Treg ratio is commonly used as an indicator of gut mucosal immunity. Here again, our results demonstrate that early ARV initiation was able to recover the memory CCR6^+^ Th17/Treg ratio only in blood and not in MLNs (Fig. 5A). To gain further insight into the dynamics of the events, we compared available matched blood and MLN specimens of early ARV-treated animals to measure Treg and CCR6^+^ Th17 cell frequencies. We observed systematically higher Treg frequencies and lower memory CCR6^+^ Th17 frequencies and CCR6^+^ Th17/Treg ratios in the MLNs compared to blood (Fig. 5B). We and others have previously reported that the tryptophan-catalyzing enzyme IDO-1 is involved in the degradation of RORγt, the Th17 cell transcription factor, resulting in changes in the Th17/Treg balance in favor of Tregs, in line with SIV/HIV disease progression (24, 25, 37). In line with the lack of significant improvement in the memory CCR6^+^ Th17/Treg ratio, no effect of early ARV initiation was observed on IDO-1 expression in MLNs, and a negative correlation was observed between total Treg (CD4^+^ FoxP3^+^) frequencies and RORγt mRNA expression (Fig. 5C and D). Overall, these results indicated that early ARV initiation reduced Treg frequencies and significantly improved the memory CCR6^+^ Th17/Treg ratio only in the blood and not in the MLNs of SIV-infected RMs.

### Early ARV initiation decreases Treg thymic output but does not affect Treg accumulation within the MLNs.

**FIG 6:**
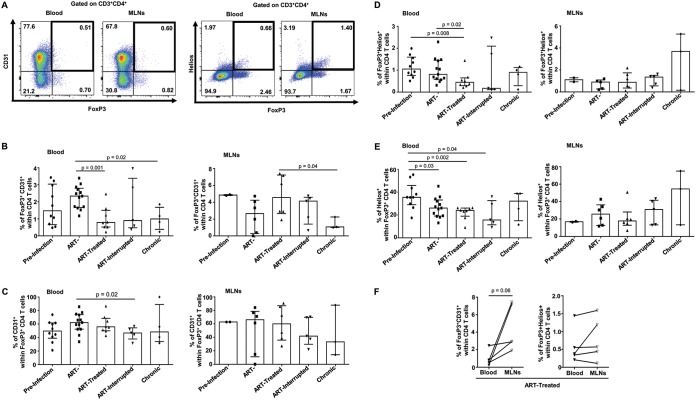
Effect of early ARV initiation on Treg thymic output. (A) Gating strategy used in flow cytometry to define thymic Tregs in both whole blood and MLNs. Recently thymic migrated Tregs were defined as FoxP3^+^ CD31^+^ and Helios^+^ FoxP3^+^ CD4^+^ T cells. (B) Percentages of CD31^+^ FoxP3^+^ CD4 T cells among total CD4 T cells in whole blood and MLNs. (C) Percentages of CD31^+^ cells among FoxP3^+^ CD4 T cells in whole blood and MLNs. (D) Percentages of Helios^+^ FoxP3^+^ CD4 T cells among total CD4 T cells in whole blood and MLNs. (E) Percentages of Helios^+^ cells among FoxP3^+^ CD4 T cells in whole blood and MLNs. (F) Differential frequencies of CD31^+^ and Helios^+^ Tregs in matched blood versus MLNs of early ARV-treated animals.

**FIG 7:**
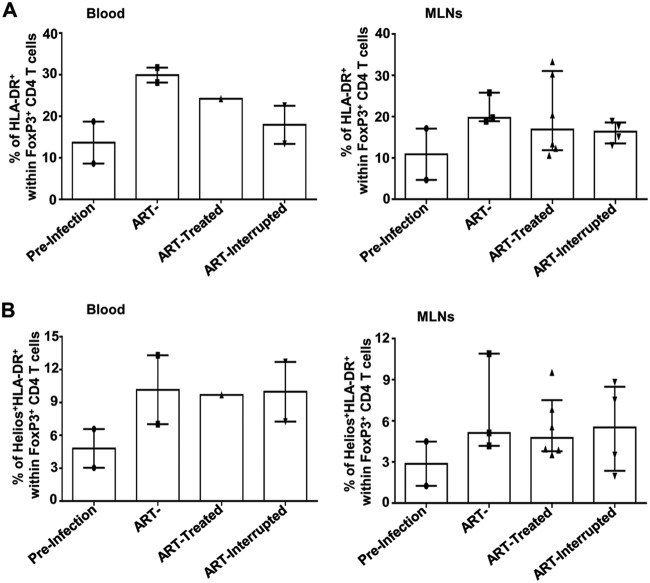
Levels of immune activation of Tregs during acute infection and following ARV initiation. (A) Percentages of HAL-DR^+^ cells among FoxP3^+^ CD4 T cells in whole blood and MLNs. (B) Percentages of Helios^+^ HAL-DR^+^ cells among FoxP3^+^ CD4 T cells in whole blood and MLNs.

Tregs may originate either directly from the thymus (thymic Tregs) or from naive CD4 T cells in the context of inflammation (14). To evaluate the origin of Treg generation and to explain the differences in Treg dynamics between blood and MLNs, we assessed the expression of CD31 and Helios on Tregs. CD31 is a marker for recently migrated CD4 T cells from the thymus (37), whereas Helios is a specific marker of thymic Tregs (38, 39). Only in blood did SIV infection induce a rapid increase in the frequency of recently thymic migrated CD31^+^ Tregs during the acute phase compared to uninfected RMs; this effect was normalized following early ARV treatment. In contrast to blood, a decrease in the frequencies of CD31^+^ Tregs was observed in the MLNs (Fig. 6A to C), which follows the total CD4 dynamics within MLNs, as shown in the Fig. 1. We then showed a significant decrease in Helios expression by Tregs in the blood of treated SIV-infected RMs versus untreated RMs in the acute phase of infection. Of interest, we observed that the frequencies of Helios^+^ Tregs remained unchanged in MLNs regardless of ARV initiation (Fig. 6A, D, and E). In treated animals, we observed higher frequencies of CD31^+^ Tregs in the MLNs versus blood but no difference in Helios^+^ Tregs between the two compartments (Fig. 6F), suggesting that in MLNs, Tregs are differentiated locally from newly generated thymic CD4 T cells, which are homing to this tissue. In agreement with this observation, we also documented higher Treg activation (HLA-DR^+^) in the MLNs of SIV-infected animals, which were not affected by early ARV treatment (Fig. 7A). In addition, a very small portion of Tregs coexpress Helios and HLA-DR (Fig. 7B).

### Early ARV treatment decreases the frequencies of CTLA-4^+^ PD-1^–^ memory CD4 T cells only in blood.

**FIG 8:**
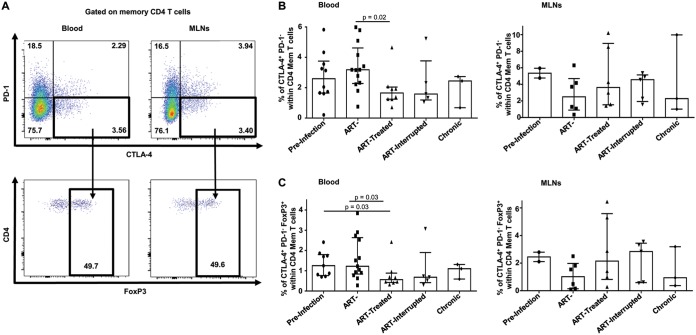
Effect of early ARV initiation on CTLA-4^+^ PD-1^–^ and FoxP3^+^ CTLA-4^+^ PD-1^–^ CD4 memory T cells. (A) Gating strategy used in flow cytometry to define CTLA-4^+^ PD-1^–^ and FoxP3^+^ CTLA-4^+^ PD-1^–^ CD4 memory T cells in both whole blood and MLNs. (B) Percentages of CTLA-4^+^ PD-1^–^ cells among memory CD4 T cells in whole blood and MLNs. (C) Percentages of FoxP3^+^ CTLA-4^+^ PD-1^–^ T cells among memory CD4 T cells in whole blood and MLNs.

Recently, a new subset of memory CD4^+^ T cells expressing CTLA-4^+^ PD-1^–^ that shares characteristics with Tregs contributing to viral persistence in lymph nodes has been described (21). Here, we observed that SIV infection induced both peripheral blood CTLA-4^+^ PD-1^–^ CD45RA^–^ CD4^+^ and FoxP3^+^ CTLA-4^+^ PD-1^–^ CD45RA^–^ CD4^+^ cells during the acute and chronic phases of SIV infection (Fig. 8). Early ARV initiation significantly decreased the level of these subsets, reaching normal frequencies compared to healthy RMs. In contrast to peripheral blood, the frequencies of both populations remained unchanged in the MLNs within all study groups. Therefore, early ARV initiation decreased the levels of CTLA-4^+^ PD-1^–^ Tregs only in peripheral blood and not in the MLNs of SIV-infected RMs.

### TGF-β1 and collagen-1 mRNA expression persists in MLNs despite early ARV treatment.

**FIG 9:**
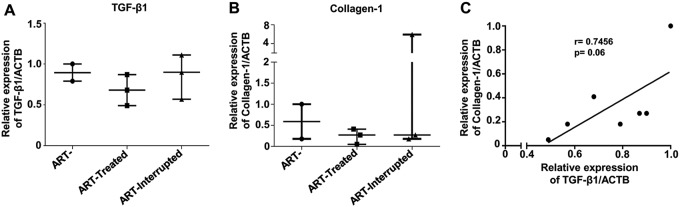
Effect of early ARV initiation on mRNA expression of markers of MLN fibrosis in SIV-infected RMs. Quantitative RT-PCR was used to quantify mRNA expression of TGF-β1 (A) and collagen-1 (B) relative to the ACTB housekeeping gene within the MLNs. (C) Correlation between TGF-β1 and collagen-1 mRNA expression within the MLNs.

It is well known that TGF-β1 production by Tregs in mucosal tissues starts very rapidly after SIV/HIV infection, resulting in collagen deposition and tissue fibrosis; this is one of the main obstacles in the recovery of gut mucosal immunity following ARV (23, 28, 40). Our results showed that early ARV initiation did not improve the expression of the GALT fibrosis markers TGF-β1 and collagen-1 mRNA within the MLNs (Fig. 9A and B). Accordingly, we observed a positive trend between TGF-β1 and collagen-1 expression (Fig. 9C).

## DISCUSSION

It is well known that GALT represents a major site of SIV/HIV pathogenesis and immune dysfunction during suppressive ARV treatment (2, 41). Increased frequencies and the immunosuppressive functions of Tregs are hallmarks of SIV/HIV infections, and these cells are involved in GALT fibrosis via TGF-β1 production, as well as the inhibition of antivirus-specific immune responses (13, 14, 42, 43). The initiation of ARV treatment upon viral exposure or diagonosis is recommended in clinical practice, since it results in a better CD4 recovery and nadir, a smaller reservoir size, and a reduction in immune activation (2, 27). Here, for the first time, we assessed the impact of early ARV initiation at day 4 postinfection on Treg dynamics in the MLNs versus the blood of RMs. The early time point of ARV initiation at day 4 has been previously shown in RMs to suppress peripheral VL, while SIV DNA has been already seeded within their MLNs (44). Our results demonstrated that, despite improvements in the levels of immune activation and recovery of memory CCR6^+^ Th17 cells in both blood and MLNs after early ARV initiation, the dynamics of the Tregs were distinct. We observed the accumulation of total Tregs and various subsets of Tregs, including CD39^+^ Tregs, thymic Tregs, and CTLA-4^+^ PD-1^–^ Tregs, remained unchanged within the MLNs compared to blood. Accordingly, early ARV initiation failed to decrease the expression of TGF-β1 and collagen-1 as markers of GALT fibrosis.

SIV/HIV infections drive generalized immune activation, even under suppressive ARV treatment (8, 30, 41, 45). In addition to HLA-DR as a marker of T-cell activation, the ectonucleotidase CD39 is a functional marker of T-cell activation and, in concert with CD73, hydrolyzes inflammatory ATP into ADP and finally into immunosuppressive adenosine (32, 46). CD39/adenosine pathways have been reported by us and others to be involved in disease progression during SIV/HIV infections (33, 35, 47). As expected, we observed higher levels of T-cell immune activation in untreated SIV-infected RMs during both acute and chronic phases of infection. Early ARV initiation decreased the levels of immune activation considerably, along with a decline in the peripheral viral load. Our results are in line with previous reports about the effect of early treatment during the acute phase on the control of immune activation levels in the periphery and in the GALT of HIV-infected individuals (48–50). In HIV infection, ARV are able to partially to completely normalize the frequencies of Tregs (19, 51, 52), but the effect of very early ARV initiation on Treg dynamics, specifically within MLNs, has been understudied. Consistent with previous reports, we observed an increase in both total Tregs and CD39^+^ Tregs in the blood of SIV-infected animals in acute infection and an even greater increase in chronic stages of infection. Importantly, in contrast to peripheral blood, early ARV initiation failed to decrease the levels of total Tregs and CD39^+^ Tregs, and even a slight increase in the percentages of these populations was observed in MLNs. This could be due to the migration of CCR6^–^ Tregs into the GALT following ARV initiation, as previously reported in chronic HIV infection (53).

The accumulation of Tregs and higher levels of TGF-β1 within the GALT results in tissue fibrosis via collagen-1 deposition, which begins in the acute phases of SIV/HIV infections (23, 40). Importantly, GALT fibrosis occurs even in HIV elite controllers, despite their natural suppression of the viremia (26). We showed that early ARV treatment did not impact the levels of both TGF-β1 and collagen-1 mRNA expression, and a strong positive correlation was observed between TGF-β1 and collagen-1 expression. Importantly, we have previously reported that in the GALT, TGF-β1 impairs the cytotoxic functions of CD8 T cells and induces their apoptosis, resulting in viral dissemination within the MLNs (4). Therefore, while this remains to be confirmed by immunohistochemistry, our results suggest a link between persistent Treg homing in the MLNs and consequently TGF-β1 production and collagen deposition within the MLNs, despite a decrease in T-cell immune activation after early ARV initiation.

Several sudies have shown that memory CCR6^+^ Th17 cells are highly permissive to SIV/HIV infections, and a rapid depletion of these cells happens rapidely following infection (5–7, 11, 54). CCR6, the CCL20 and β-defensin receptor, regulates the migration of T cells to the GALT (55) and is widely used as a marker of Th17 cells (5, 11, 41). The Th17/Treg ratio is frequently used as an indicator of GALT immunological functions. Our results showed that early ARV initiation restored the levels of memory CCR6^+^ Th17 cells in both the periphery and the MLNs, even at higher levels than in noninfected animals. These dynamics have been also confirmed by the assessment of IL-17a-producing cells from the MLNs. This was expected since it has been already demonstrated that ARV treatment restores CD4^+^ T memory stem cell homeostasis in SIV-infected RMs (56). Accordingly, following early ARV initiation, the memory CCR6^+^ Th17/Treg ratio was only restored in blood but remained unchanged in the MLNs in line with the accumulation of Tregs in this tissue. Of particular importance, in matched specimens from early ART-treated animals, systematic higher Treg frequencies and lower memory CCR6^+^ Th17 frequencies were observed in the MLNs compared to blood, which demonstrates clearly their differential dynamics in these tissues. A recent study showed that the later initiation of ARV treatment at 6 weeks postinfection was not able to recover the Th17/Treg ratio in the GALT during the acute SIV infection of RMs (57). The consistent increased frequencies of Tregs in MLNs could be linked to IDO-1 enzyme expression (4). IDO-1 catabolizes tryptophan into immunosuppressive kynurenine and promotes a Th17/Treg imbalance in favor of Tregs via the degradation of RORγt during HIV infection, as reported by us and others (18, 58, 59). In addition, IDO-1 is highly expressed by macrophages, and accumulation of these cells in the GALT and MLNs has been reported during SIV infection (4, 60). Our results showed that, along with the subsequent increase in Tregs in MLNs, IDO-1 mRNA expression was not decreased following early ARV treatment. Altogether, these findings demonstrate that despite decreased immune activation and recovery of memory CCR6^+^ Th17 cells, the accumulation of Tregs within the MLNs seems to be not reversible.

To better evaluate the origin of Tregs in MLNs, we assessed the expression of both CD31 and Helios as markers of recently thymic migrated CD4 T cells and thymic Tregs, respectively (37–39). We observed an increase in CD31^+^ Tregs during acute SIV infection in the absence of ARV in line with viral load and immune activation, whereas ARV normalized the frequencies of recently migrated CD31^+^ Tregs in contrast to their dynamics in the MLNs. It has been reported that Helios^+^ Tregs proliferate more *in vivo* and produce higher levels of TGF-β1 compared to Helios^–^ Tregs (61), and the expression of Helios^+^ Tregs in blood was correlated with the control of HIV replication (62). Accordingly, we demonstrated that the frequencies of thymic Tregs were decreased only in the blood after early ARV initiation, whereas the frequencies of Helios^+^ Tregs remained unchanged following SIV infection. This observation, together with the higher frequencies of CD31^+^ Tregs but not of Helios^+^ Tregs in the MLNs, suggests that Tregs within the MLNs could be differentiated locally form the newly generated thymic CD4 T cells, in agreement with the recovery of total CD4 T cells following ART. Our study also demonstrated that SIV infection increased the frequencies of both CD4^+^ memory CTLA-4^+^ PD-1^–^ and FoxP3^+^ CTLA-4^+^ PD-1^–^ T cells in SIV-infected RMs; these frequencies were normalized by early ARV initiation only in the blood. These CTLA-4^+^ PD-1^–^ subsets share Treg characteristics and were reported to contribute to viral persistence within the peripheral lymph nodes of ARV-treated SIV-infected RMs (21). Here, we extended this initial observation by demonstrating the persistence of this population in the MLNs despite early ARV treatment. Although our findings remain to be confirmed in further studies, these findings suggest that at least a subset of Tregs might contribute to the viral persistence in the GALT despite very early ARV initiation.

In summary, here, we present evidence indicating differential dynamics of Tregs in the blood and MLNs of SIV-infected RMs following early ARV initiation. Indeed, despite the beneficial impact of early ARV initiation within the first few days of SIV infection on immune activation and memory CCR6^+^ Th17 cells in both blood and MLNs, the frequencies and homing of various subsets of Tregs within the MLNs, as well as markers of tissue fibrosis, were not improved. These findings highlight the role of Tregs in SIV/HIV pathogenesis in GALT and represent Tregs as potential targets for further immunotherapeutic strategies.

## MATERIALS AND METHODS

### Ethics statement.

All RMs were housed at the nonhuman primate facilities of the Laval University, Quebec City, Quebec, Canada, in accordance with the rules and regulations of the Canadian Council on Animal Care (http://www.ccac.ca). This protocol was approved by the Laval University Animal Protection Committee (no. 106004). Animals were fed by standard monkey chow diet supplemented daily with fruit and vegetables and water *ad libitum*. Social enrichment was delivered and overseen by a veterinary staff, and overall animal health was monitored daily. Animals showing significant signs of distress, disease, and weight loss were evaluated clinically and were humanely euthanized, using an overdose of barbiturates, according to the guidelines of the Veterinary Medical Association.

### Animals, infection protocol, and ARV cocktail.

A total of 32 female RMs, seronegative for SIVmac, STLV-1 (simian T leukemia virus type 1), SRV-1 (type D retrovirus), and herpes B viruses were enrolled in this study (Table 1). A total of 25 animals were infected intravenously with 20 50% animal infectious doses of SIVmac251 virus, and the specimens were collected in both acute and chronic phases of infection. To evaluate the effect of early ARV initiation, seven monkeys were treated 4 days after the infection in a daily manner with an ARV cocktail that included tenofovir (20 mg/kg), emtricitabine (20 mg/kg), indinavir (2 mg/kg), ritonavir (20 mg/kg), and raltegravir (20 mg/kg). After 8 weeks of ARV treatment, the therapy was interrupted in three RMs. Two groups of nontreated animals in both acute (*n* = 13) and chronic (*n* = 4) phases were also assessed. To assess the uninfected baseline status, blood specimens were obtained from 10 animals 3 days before the SIVmac251 infection. MLN specimens from two uninfected animals were also assessed to establish a baseline status in the MLNs.

### Cell isolation.

At the indicated time points (Table 1), peripheral blood and MLNs were collected upon euthanasia. Blood specimens were collected in EDTA tubes, and staining for flow cytometry analysis was performed using whole blood. MLNs were mechanically disrupted and grinded over a 70-μm cell strainer to avoid any collateral effect of enzymatic digestion by collagenase or other proteases on the expression of surface markers. The whole blood specimens obtained without euthanasia were frozen, and flow cytometric analyses were performed in batch on frozen samples.

### *Ex vivo* flow cytometric analysis.

Multicolor flow cytometry was performed using whole blood and MLN cells. Predetermined optimal concentrations of fluorochrome-conjugated antibodies were used for the staining, as detailed in Table 2. Dead cells were excluded from the analysis using a Live/Dead Fixable Aqua dead cell stain kit (Invitrogen). After the extracellular staining, FoxP3, Helios, and CTLA-4 staining was performed following permeabilization with a transcription factor buffer set (BD Bioscience). Flow cytometry acquisition was performed on a three-laser BD Fortessa-X20 cytometer, and results were analyzed by using FlowJo v10.2.

**TABLE 2:** Monoclonal antibodies used for flow cytometry analysis

MAb-fluorochrome	Clone	Source company	Catalog no.
CD3-Alexa Fluor-488	SP34.2	BD Pharmingen	557705
CD3-Alexa Fluor-700	SP34.2	BD Pharmingen	557917
CD4-BV650	L200	BD Horizon	563737
CD8-APC-R-700	SK1	BD Horizon	565192
HLA-DR-BV605	G46-6	BD Pharmingen	562844
CD31-APC-cy7	WM59	BD Pharmingen	563653
CD39-FITC	eBioA1	eBioscience	11-0399-42
CD39-APC	eBioA1	eBioscience	17-0399-42
CD45RA-APC-H7	5H9	BD Pharmingen	561212
CD152 (CTLA-4)-APC	BNI3	BD Pharmingen	555855
CD196 (CCR6)-PE	11A9	BD Pharmingen	551773
CD279 (PD-1)-BV711	EH12.2H7	BioLegend	329928
FoxP3-PE-CF594	236A/E7	BD Horizon	563955
Helios-Pacific Blue	22F6	BioLegend	137220
IFN-γ-PE-cy7	B27	BD Pharmingen	557643
IL-17A-APC	eBio64DEC17	eBioscience	17-7179-41

### IL-17A production following *in vitro* stimulation.

Isolated cells from MLNs were stimulated with phorbol myristate acetate (PMA) at 50 ng/ml and ionomycin (1 μg/ml) for 4 h, followed by incubation in the presence of brefeldin A and monensin (both from BD Bioscience) for 4 h. The intracellular production of IL-17a and gamma interferon (IFN-γ) was assessed by flow cytometry.

### Viral RNA quantification.

Viral loads in the sera of SIV-infected RMs were quantified by quantitative real-time PCR (RT-PCR) using a PureLink viral RNA/DNA kit (Invitrogen). A plasmid encoding the *gag* gene of SIVmac251 was used as a standard. Amplifications were carried out with a 7500 real-time PCR system (Applied Biosystems) using the following parameters: 50°C/5 min, 95°C/20 s, and 40 cycles of 95°C/15 s and 60°C/1 min. Samples were run in duplicates, and results are expressed as SIV RNA copies/ml, with a limit of detection of 40 copies/ml (63).

### Quantification of mRNA expression by RT-PCR.

Total RNA was extracted from snap-frozen MLN tissues obtained upon euthanasia using an AllPrep DNA/RNA/miRNA Universal kit (Qiagen, Germany). cDNA was then synthetized using M-MLV reverse transcriptase (Invitrogen) on total RNA (140 ng) according to the manufacturer’s protocol. The specific primers used for the detection of TGF-β1, collagen-1, IDO-1, RORγt, and housekeeping gene ACTB are described in Table 3. Reactions were done in duplicate using a LightCycler 480 Instrument II (Roche, Switzerland) with a LightCycler 480 SYBR green I Master (Roche, Germany). The cycling conditions were as follows: 5 min at 95°C; 40 amplification cycles of 15 s at 95°C, 20 s at 59°C, and 20 s at 72°C; 1 cycle of 5 s at 95°C and 30 s at 65°C; and finally 10 s at 40°C. We generated a negative control without cDNA to confirm that no contamination occurred. The negative control yielded no amplification or a crossing point (Cp) value up to or equal to 35. The expression of each gene was quantified relative to the housekeeping gene ACTB.

**TABLE 3:** Primers used in RT-PCRs

Gene	Sequence (5′–3′)	
	Forward	Reverse
ACTB	CAGCAAGCAGGAGTATGACG	AAGTCACAGTCCGCCTAGAA
RORγt	CGAGATGCTGTCAAGTTCGG	CCCAATGTGTAGGTGAGGGT
TGF-β1	GTGGGATACTGAGACACCCC	CCTCGAGGGAAAGTTGAGGT
IDO-1	GGTCTGGTGTATGAGGGGTT	CAAACTCACGGACTGAGGGA
COL-1	ATCACCCACCGACCAAGAAA	GGCCAGGTTAGAGAAGGGAG

### Statistical analysis.

Prism v6.01 (GraphPad, San Diego, CA) was used for statistical analyses. The results are presented as medians with interquartile ranges. Differences among the different groups of animals were determined by using a Mann-Whitney rank test for unpaired variables. A Pearson rank correlation test was used to identify the associations among study variables. A Wilcoxon matched-pair signed-rank test was used to compare paired variables. Statistical significances (*P* ≤ 0.05) are indicated in the figures.
